# Normalizing JMJD6 Expression in Rat Spinal Dorsal Horn Alleviates Hyperalgesia Following Chronic Constriction Injury

**DOI:** 10.3389/fnins.2018.00542

**Published:** 2018-08-07

**Authors:** Cheng Mo, Mengyuan Xu, Cen Wen, Ruimin Chang, Changsheng Huang, Wangyuan Zou, Xiaoyan Zhu, Qulian Guo

**Affiliations:** ^1^Department of Anesthesiology, Xiangya Hospital of Central South University, Changsha, China; ^2^Department of Anesthesiology, The People's Hospital of Guangxi Zhuangzu Autonomous Region, Nanning, China; ^3^Department of Thoracic Surgery, Xiangya Hospital Central South University, Changsha, China

**Keywords:** JMJD6, HIF-1α, chronic constriction injury, neuropathic pain, caspase-3

## Abstract

Jumonji domain-containing protein 6 (JMJD6) is a homolog of hypoxia-inducible factor (HIF) asparaginyl hydroxylase, an inhibitor of HIF. HIF-1α is known to participate in neuropathic pain (NPP) during chronic constriction injury (CCI); however, the roles of JMJD6 in NPP have not been systematically investigated. In this study, we examined the temporal distribution and cellular location of JMJD6 in the spinal cord during CCI. In addition, we assessed behavioral changes representative of NPP in rats. Following CCI, lentiviral vectors (LV-JMJD6) were intrathecally administered to observe the changes in the expression of JMJD6, HIF-1α, and its downstream factor caspase-3. Co-immunoprecipitation was used to detect potential interactions between JMJD6 and HIF-1α. We found that JMJD6 was decreased in rats following CCI, which was accompanied by significant NPP–associated behavioral changes. JMJD6 was mainly expressed in neurons. Intrathecal injection of LV-JMJD6 following CCI alleviated the thermal and mechanical hyperalgesia, normalized JMJD6 protein expression, and decreased HIF-1α protein expression with a corresponding reduction in caspase-3 protein expression. Furthermore, the co-immunoprecipitation analyses showed that JMJD6 and HIF-1α protein immunoprecipitated with each other, indicating an interaction between these two proteins. Taken together, the results suggest that JMJD6 may serve as a sensor in neurons of the adult rat spinal cord during the CCI state. Furthermore, JMJD6 may exert its function in NPP by regulating HIF-1α in rats exposed to CCI.

## Introduction

A growing population worldwide are suffering symptoms of neuropathic pain (NPP), such as allodynia, hyperalgesia, or paresthesia, which are caused by lesions in the somatosensory nervous system (Treede et al., 2008; Geber et al., 2009; Haanpää et al., 2010). Many new therapies have been proposed, such as voltage-gated sodium or calcium channel blockers; however, there is currently a lack of effective and specific treatment options, which indicates that advancing our knowledge of the pathophysiology and the mechanisms underlying this type of pain is a priority (Chang et al., 2014; Schestatsky et al., 2014; Schug and Goddard, 2015; Nascimento et al., 2016).

Jumonji domain-containing protein 6 (JMJD6) is a member of the JMJD family that mediates the demethylation of histone H3 and H4 at arginine 2 and 3, respectively (Chang et al., 2007). JMJD6 and bromodomain-containing protein 4 mediate both RNA demethylase and histone activity on anti-pause enhancers (Liu et al., 2013). Recently, it has been shown that JMJD6 exerts its inhibitory function through the demethylation of H4R3me2a, leading to suppression of Myc-induced apoptosis (Aprelikova et al., 2016). Other reports have demonstrated that the level of histone methylation influences the synaptic plasticity in the nervous system (Duman and Newton, 2007; Ng et al., 2009; Füllgrabe et al., 2011; Jakovcevski et al., 2015). Tsai et al. (2012) have reported that thermal hyperalgesia is induced when histone methylation is increased in the spinal cord. Therefore, increasing the level of histone demethylation *via* drugs relieved hyperalgesia, further indicating that histone demethylation may help to relieve the pathologic process of NPP (Tsai et al., 2012).

Hypoxia-inducible factor 1 (HIF-1) is a dimer that is composed of an α- (the active center) and a β-subunit. HIF-1 is a central regulator of oxygen homeostasis in mammals (Wang and Semenza, 1995; Loboda et al., 2010). Sequence analyses have suggested that JMJD6 is a homolog of the HIF asparaginyl hydroxylase, a HIF-1 inhibitor (Semenza, 2000). Recently, HIF-1α has been associated with NPP in an adult rat model of chronic constriction injury (CCI) of the sciatic nerve (Hsieh et al., 2012). Caspase-3 is a target gene of HIF-1α (Van Hoecke et al., 2007; Chen et al., 2009); HIF-1 functionally binds to the caspase-3 promoter after photothrombotic cerebral ischemia (Van Hoecke et al., 2007).

It is unknown whether JMJD6 exerts a role in NPP through HIF-1α. In this study, we sought to detect the temporal distribution and cellular location of JMJD6 and observe any changes in JMJD6, HIF-1α, and caspase-3 expression after JMJD6 was normalized in CCI rats. In addition, we observed the potential interaction between JMJD6 and HIF-1α *via* co-immunoprecipitation analyses.

## Materials and methods

### Animals and the CCI model

All animal-related operations were carried out following the National Institute of Health guide on the care and use of laboratory animals. All protocols were approved by the Animal Care Committee of Central South University and were in accordance with the guidelines provided by the National Institute of Health. Special care was taken to avoid animal suffering and use the minimum number of animals necessary for statistical validity.

Sprague Dawley rats (220–250 g, male) were purchased from the Animal Experiment Center of Xiangya School of Medicine in Central South University. Rats were anesthetized using isoflurane (1–3%) prior to the initiation of surgical procedures. CCI surgery was performed as described in a previous study (Bennett and Xie, 1988). Briefly, four snug ligatures were placed on the left sciatic nerve (4–0 chromic gut) at an interval of 1 mm. The left sciatic nerve of rats in the sham group was exposed but no ligatures were applied. All surgical procedures were performed by the same individual to minimize the variability.

### Behavioral tests

Rats underwent behavioral tests before CCI and on days 3, 7, 14, and 21 post-CCI. Only rats showing thermal hyperalgesia and mechanical allodynia in the CCI group were used in the following studies. The ipsilateral thermal withdrawal latency (TWL) was measured using a Hargreaves Tes7370 (Ugo Basile, Comerio, Italy), as described in a previous study (Xu et al., 2018). The mechanical withdrawal threshold (MWT) was measured using an Electronic von Frey Anesthesiometer 2390 (IITC Life Science, USA), as previously described (Cunha et al., 2008; Zhu et al., 2012).

### Lentiviral vectors

The plasmid (GV358, Ubi-MCS-3FLAG-SV40-EGFP-IRES-puromycin) was used to create the JMJD6 lentiviral vector, LV-JMJD6. The sequence for the mRNA targeting the rat *Jmjd6* gene (GenBank ID: NM-001012143) was acquired. In order to get the target gene segment, a PCR primer was designed as follows: *Jmjd6* (1,253 bp), sense sequences, 5′-GAGGATCCCCGGGTACCGGTCGCCACCATGAACCACAAGAGCAAGAAGC-3′; antisense sequences, 5′-TCCTTGTAGTCCATACCCCTGGAGGAACTGCGCTCTTTG-3′. PrimeSTAR HS DNA polymerase (cat. no. R010B, Takara Biotechnology Co., Ltd., Dalian, China) and primers (GeneRay Biotech Co., Ltd., Shanghai, China) were used for polymerase chain reaction (PCR) amplification of *Jmjd6* cDNA. The GV358, pHelper 1.0 and pHelper 2.0 vectors were used as the lentivirus vectors (Shanghai GeneChem Co., Ltd., Shanghai, China). The Plasmid DNA Extract kit (Qiagen, Inc., Valencia, CA, USA) was used to extract plasmid DNA. These oligonucleotides were constructed into the plasmid (GV358, Ubi-MCS-3FLAG-SV40-EGFP-IRES-puromycin). The same plasmid not expressing JMJD6 was used as a negative control virus (LV-NC) (Shanghai GeneChem Co., Ltd.). The recombinant vector and packaged plasmids were co-transduced into 293T cells (American Type Culture Collection, Manassas, VA, USA). The recombinant virus titer was 1 × 108 TU/ml.

### Intrathecal injection of lentiviral vectors

A lumbar intrathecal catheter was purchased from Anilab (Ningbo, China). Catheter implantation was performed in anesthetized rats (1–3% isoflurane). The lumbar intrathecal catheter was implanted into the subarachnoid space as previously described (Ding et al., 2017). The success of catheter insertion was judged using the intrathecal lidocaine test. Only rats that displayed paralysis within 30 s followed by total recovery after 30 min were considered to have a successfully implanted lumbar intrathecal catheter. Rats undergoing CCI-induced neuropathy received LV-JMJD6, LV-NC, or 0.9% saline (20 μl) through the intrathecal catheter on day 7 post-CCI surgery. Rats that underwent a sham operation were injected with 0.9% saline (20 μl). Following the virus administration, the catheter was flushed with 0.9% saline(10 μl).

### Sample preparation and western blot analysis

Rats under deep anesthesia were perfused with 0.9% NaCl through the ascending aorta. When the discharge liquid became transparent, the lumbar spinal cords were removed immediately. Ipsilateral L4–6 spinal cord segments were prepared for extraction of total protein. A 10% SDS-PAGE gel was used to separate the proteins (each sample = 50 μg), which were then transferred to PVDF membranes. Non-fat dry milk (5%) blended with 0.05% Tween 20 and phosphate-buffered saline (PBS) was used on membranes for 1 h to block non-specific binding, followed by incubation with mouse anti-JMJD6 (1:300; sc-28348, Santa Cruz, USA), rabbit anti-HIF-1α (1:500; ab51608, Abcam), rabbit anti-caspase-3 (1:1,000; #9665, CST), or rabbit anti-GAPDH (1:5,000) primary antibodies overnight at 4°C. Membranes were then incubated with goat anti-mouse or anti-rabbit secondary antibodies for 1 h. An ECL kit (Pierce, USA) was used to visualize protein bands. Quantity One software (Bio-Rad, USA) was used for the densitometric analysis. Target protein levels were standardized to GAPDH.

### Immunohistochemistry, immunofluorescence, and histopathology

Tissue samples from L4–6 spinal cord segments were post-fixed with 4% paraformaldehyde at 4°C. After the tissue was dehydrated and embedded in paraffin, 5-μm thick coronal slices were obtained. Prior to incubation with primary antibodies, selected sections went through three stages: dewaxing, antigen retrieval, and elimination of peroxidase activity. After incubation with mouse anti-JMJD6 antibodies (1:300; sc-28348, Santa Cruz) at 4°C for 24 h, sections were incubated with biotinylated anti-mouse IgG (1:400; Vector) for 1 h. The immunoprecipitates were visualized using the Elite Vectastain ABC kit (Vector) for 2 h, followed by incubation with diaminobenzidine (DAB, Vector). Immunohistochemical images were digitally captured with a Nikon N-STORM Professional CCD Camera (Japan). Cells were graded “+” and counted as immunoreactive cells according to the % of maximal mean density level as described in a previous study (Chung et al., 2002). Mean immunoreactivity, which was calculated by double-blind cell counting, was used for subsequent statistical analyses.

Prior to and including antigen retrieval, the immunofluorescence procedures were the same as for the immunohistochemical analysis. After incubation with mouse anti-JMJD6 antibody (1:100; sc-28348, Santa Cruz) at 4°C for 24 h, sections were incubated with red dihydroxyfluorene (1:200; SA00006-3, Proteintech) for 2 h. Subsequently, the sections were incubated in the following sequence: (1) incubation with rabbit anti-neuronal nuclei (NeuN) antibody, a specific neuronal marker (1:500; ABN78, Millipore); rabbit anti-glial fibrillary acidic protein (GFAP) antibody, a specific marker for astrocytes (1:500; 04-1031, Millipore); rabbit anti-ionized calcium-binding adapter molecule 1 (Iba1) antibody, a specific marker for microglia (1:200; ab108539, Abcam); or rabbit monoclonal anti-HIF-1α antibody (1:100; ab51608, Abcam) at 4°C for 24 h. (2) Incubation with green dihydroxyfluorene (1:200; SA00006-2, Proteintech) for 2 h. Immunofluorescence images were digitally captured with a Nikon Eclipse Ti-SR microscope (Japan).

Histopathological procedures were used to evaluate on hematoxylin and eosin (H&E)-stained sections to determine the nerve cell destruction following intrathecal catheter implantation and injection of lentiviral vectors.

### Co-immunoprecipitation analysis

*Rattus norvegicus* adrenal pheochromocytoma PC12 cells were cultured at 37°C with 5% CO_2_ in complete growth medium (Dulbecco's Modified Eagle Medium, GIBCO; HyClone, GIBCO, 1% penicillin/streptomycin, 10% fetal bovine serum). Cells were collected and homogenized in Triton lysis buffer (0.2 mM phenylmethylsulfonyl fluoride; 20 mM Tris, pH 7.4; 1 mM EDTA; 10 mM NaF; 1 mM EGTA; 150 mM NaCl; 1 mM Na_3_VO_4_; 1% Triton X-100) at 4°C, centrifuged at 12,000 rpm and 4°C for 15 min, and then the precipitate was removed. Cell lysate (50 μl) was stored at −80°C for further analysis. The remaining cell lysate was incubated with rabbit anti-JMJD6 antibody (2 μg/100 μl lysate; sc-11366, Santa Cruz) and normal rabbit IgG antibody (2 μg/100 μl lysate; Santa Cruz). An additional 100 μl cell lysate was incubated with mouse anti-HIF-1α antibody (2 μg/100 μl lysate; ab463, Abcam) and normal mouse IgG antibody (2 μg/100 μl lysate; Santa Cruz) at 4°C for 24 h. Each lysate was incubated with protein A/G agarose beads at 4°C for 2–4 h (Santa Cruz). After further centrifugation (3,000 rpm, 4°C, 3 min), the supernatant was discarded, and the protein A/G agarose precipitate and the cell lysate were eluted with 5 × sample buffer (500 mM Tris, 50% glycerol, 10% SDS, pH 6.8) with dithiothreitol and denatured (95°C, 5 min) for western immunoblotting. The immunoprecipitates were loaded into a 12% polyacrylamide gel and electrotransferred to nitrocellulose membranes (Bio-Rad). The membranes were incubated with 5% non-fat dry milk solution for 1 h and then incubated with primary antibody (JMJD6, 1:1,000, Santa Cruz; HIF-1α, 1:1,000, Abcam) on a shaker at 4°C for 24 h. Following this, membranes were incubated with goat anti-rabbit/anti-mouse secondary antibody (1:3,000, Proteintech) for 1 h, and the membranes were visualized using an enhanced chemiluminescence detection kit (Pierce).

### Statistical procedures

Values are expressed as the mean ± standard error of the mean (SEM). Behavioral data were analyzed using repeated-measures two-way analysis of variance (ANOVA), followed by Holm–Sidak post hoc tests. The expression of JMJD6 and HIF-1α was determined using one-way ANOVA followed by the Levene's test. Quantitative estimations of JMJD6 and HIF-1α in immunofluorescence procedure were analyzed with Student's *t*-tests. Data were considered statistically significant if *p* < 0.05.

## Results

### CCI rats showed hyperalgesia accompanied by decreased expression of JMJD6 in the spinal cord

**Figure 1:**
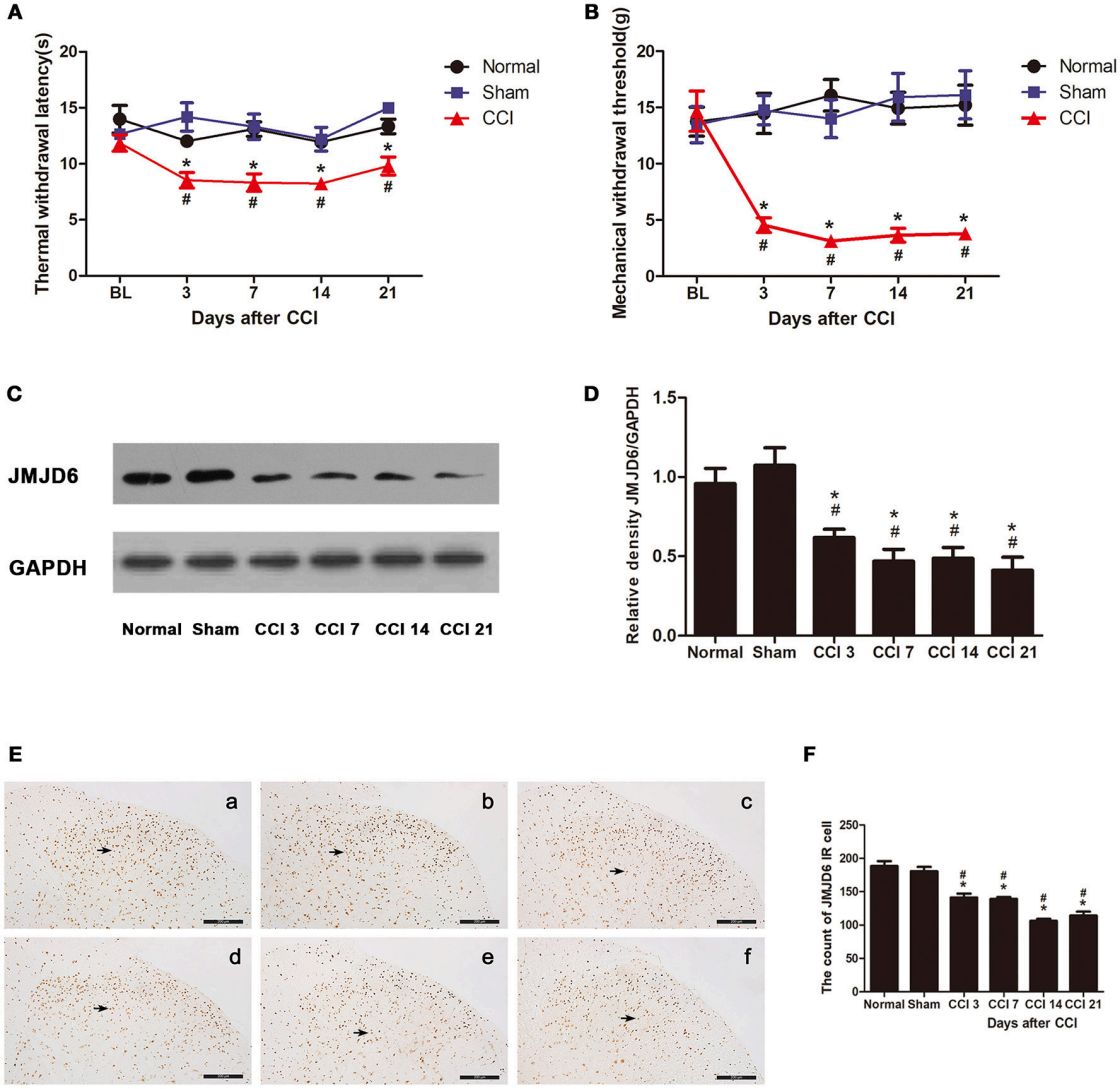
Rats show decreased expression of JMJD6 in the spinal cord accompanied by hyperalgesia after CCI surgery. **(A)** Time course of thermal withdrawal latency (TWL) in rats (mean ± SEM, *n* = 5). **(B)** Time course of mechanical withdrawal threshold (MWT) in rats (mean ± SEM, *n* = 5). **(C,D)** JMJD6 protein expression was significantly decreased in the CCI group at 3, 7, 14, and 21 days post-CCI surgery (*n* = 4). **(E)** The temporal distribution of JMJD6. Representative images of JMJD6 immunoreactivity are shown. **(a)** JMJD6 immunoreactivity in the normal group. **(b)** JMJD6 immunoreactivity in the sham-operated group. **(c–f)** JMJD6 immunoreactivity on days 3, 7, 14, and 21 post-CCI surgery. Arrows indicate representative JMJD6-positive cells. **(F)** Mean JMJD6 immunoreactivity at corresponding time points after CCI surgery, (*n* = 4). ^#^*p* < 0.05, CCI vs. normal group; *p < 0.05, CCI vs. sham group.

Rats that underwent CCI surgery showed toe closing, paw licking, and dorsiflexion, which are the representative signs of pain sensitization. As shown in Figures 1A,B, there were no significant differences in TWL and MWT between groups before CCI (*p* > 0.05). There was a decrease in TWL and MWT in rats of the CCI group on day 3 post-CCI surgery compared to the rats of the normal and sham groups (*p* < 0.05). This decrease persisted until day 21 post-CCI surgery (*p* < 0.05), which is in agreement with previous studies (Zou et al., 2011; Zhu et al., 2013).

The expression of the JMJD6 protein was significantly reduced in the CCI group at 3, 7, 14, and 21 days post-CCI in western bolt (Figures 1C,D) compared with the normal and sham groups (*p* < 0.05). The immunoreactivity of JMJD6 was mainly distributed in the nuclei in the gray matter of the spinal cord dorsal horn (Figure 1E). JMJD6 immunoreactivity was reduced in the CCI group at 3, 7, 14, and 21 days post-CCI surgery (Figures 1E,F) compared with the normal and sham groups, which matches the western blot results. This further verifies the reduction in JMJD6 protein expression following CCI.

### JMJD6 is localized in neurons but not astrocytes or microglial cells in the spinal cord following CCI

**Figure 2:**
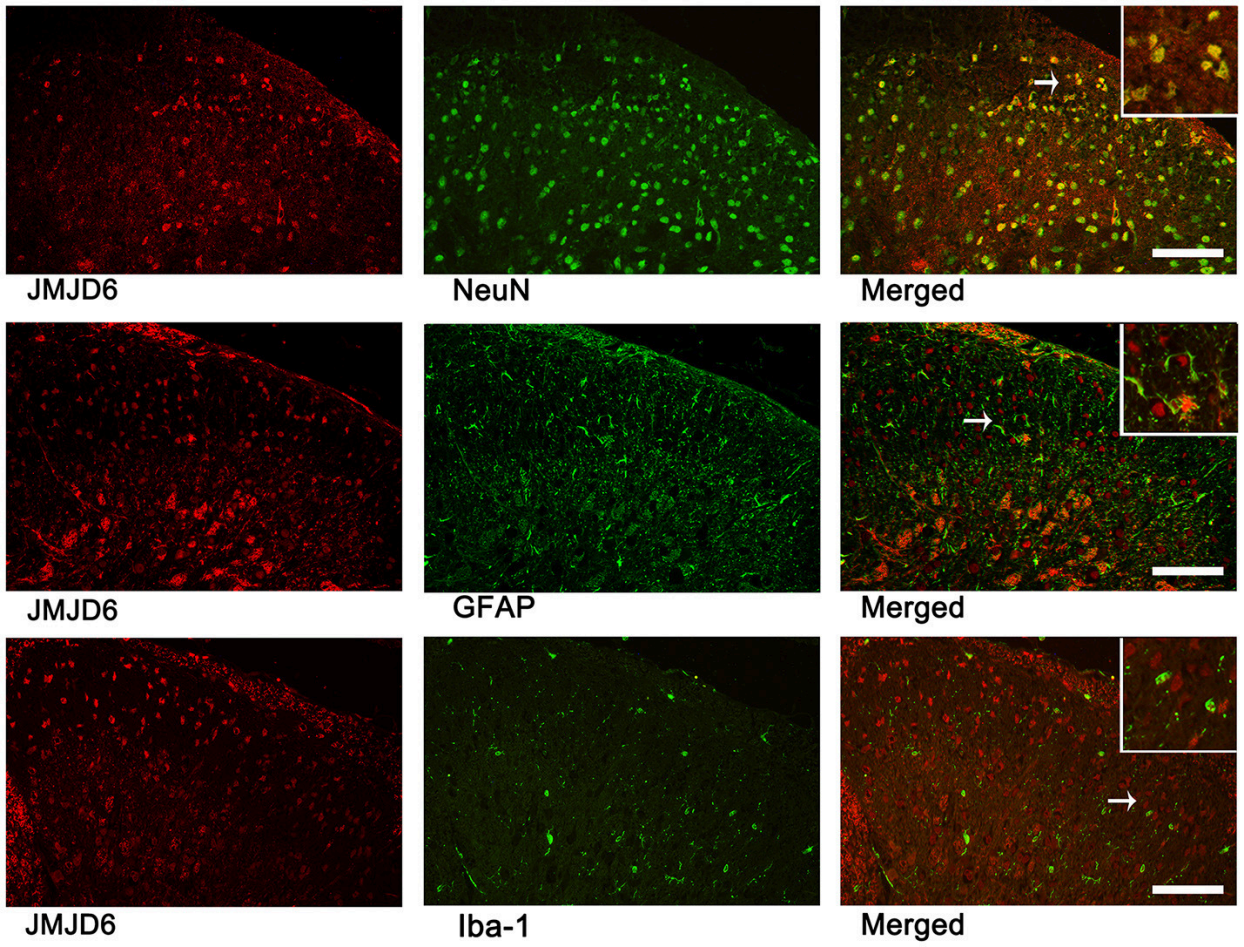
JMJD6 is expressed in neurons at day 14 following CCI surgery. Arrows indicate the cells shown at high magnification in the insets. Double immunofluorescence labeling was performed for JMJD6 (red) and NeuN (green), GFAP (green), and Iba1 (green). Scale bars = 100 μm.

We examined sections of L4–6 spinal cord on day 14 post-CCI surgery to determine the localization of JMJD6 *via* double immunofluorescence analysis (Figure 2). There were few cells double-labeled for JMJD6 and GFAP or Iba1; however, the majority of JMJD6-positive cells was also stained by NeuN. These results indicate that JMJD6, to some extent, may be more dominantly expressed in neurons following CCI in rats.

### Thermal and mechanical hyperalgesia following CCI were suppressed by intrathecal injection of LV-JMJD6

**Figure 3:**
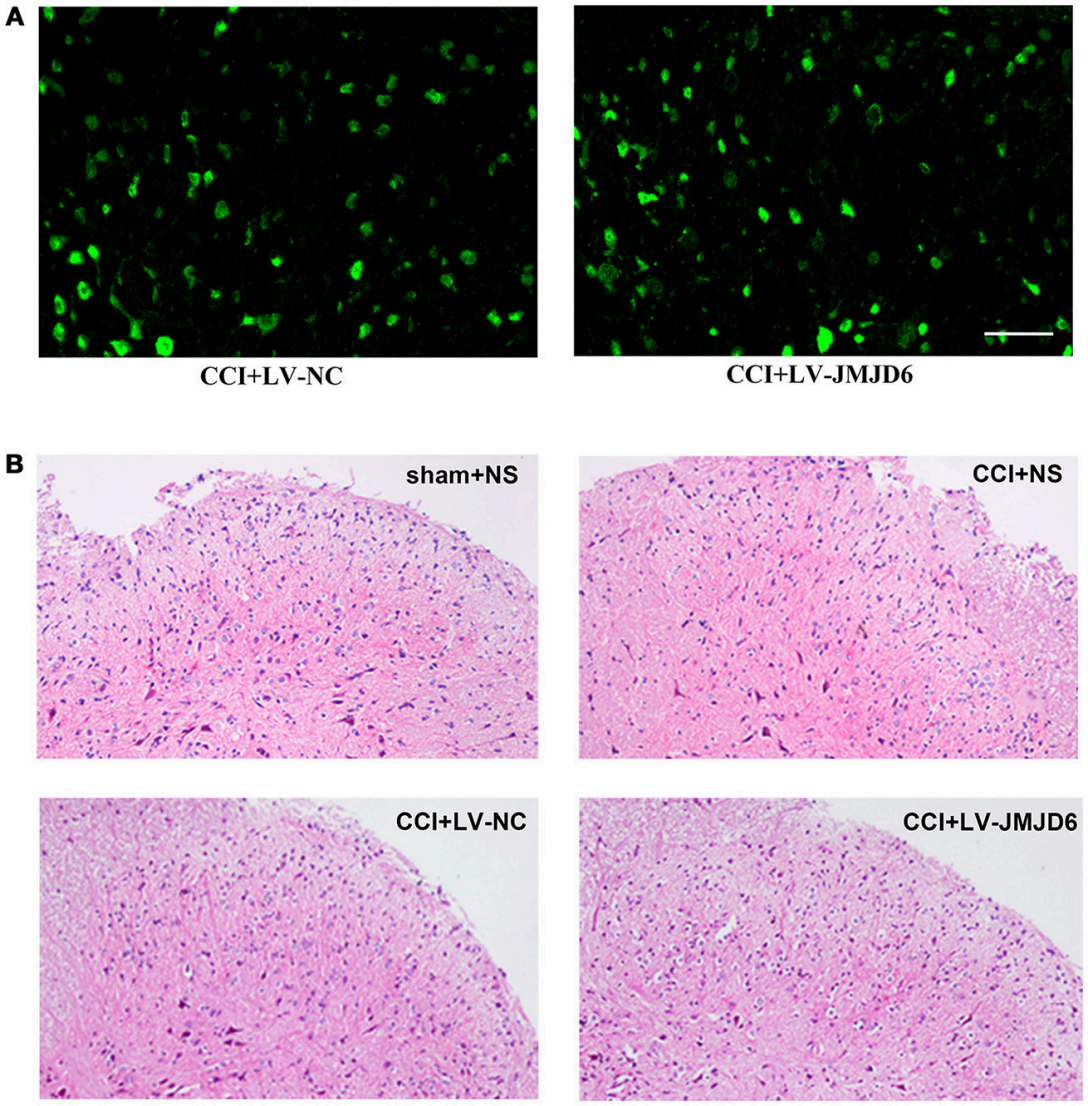
Successful intrathecal injection of lentiviral vectors. **(A)** Representative images of GFP (green) in the spinal dorsal horn of rats that received LV-NC or LV-JMJD6 lentiviral vectors. Scale bars = 50 μm. **(B)** H&E staining showing the histopathology of the spinal cord following intrathecal catheter insertion and vector injection. Sham + NS, the left sciatic nerve was only exposed + intrathecal injection with 0.9% saline. CCI + NS, CCI surgery + intrathecal injection with 0.9% saline. CCI + LV-NC, CCI surgery + intrathecal injection with LV-NC. CCI + LV-JMJD6, CCI surgery + intrathecal injection with LV-JMJD6.

First, we determined the efficacy of the lentiviral transfection system. Positive staining for enhanced green fluorescent protein (eGFP, green) in the spinal dorsal horn of rats that received an intrathecal injection of LV-JMJD6 or LV-NC revealed a successful transfection with the lentiviral vectors (Figure 3A). There was no obvious destruction of the cellular morphology or the outline of the L4–6 spinal cord between groups, as assessed by H&E staining (Figure 3B). These results suggest that intrathecal catheter implantation and injection of lentiviral vectors or NS has no obvious deleterious effects on the spinal dorsal horn.

**Figure 4:**
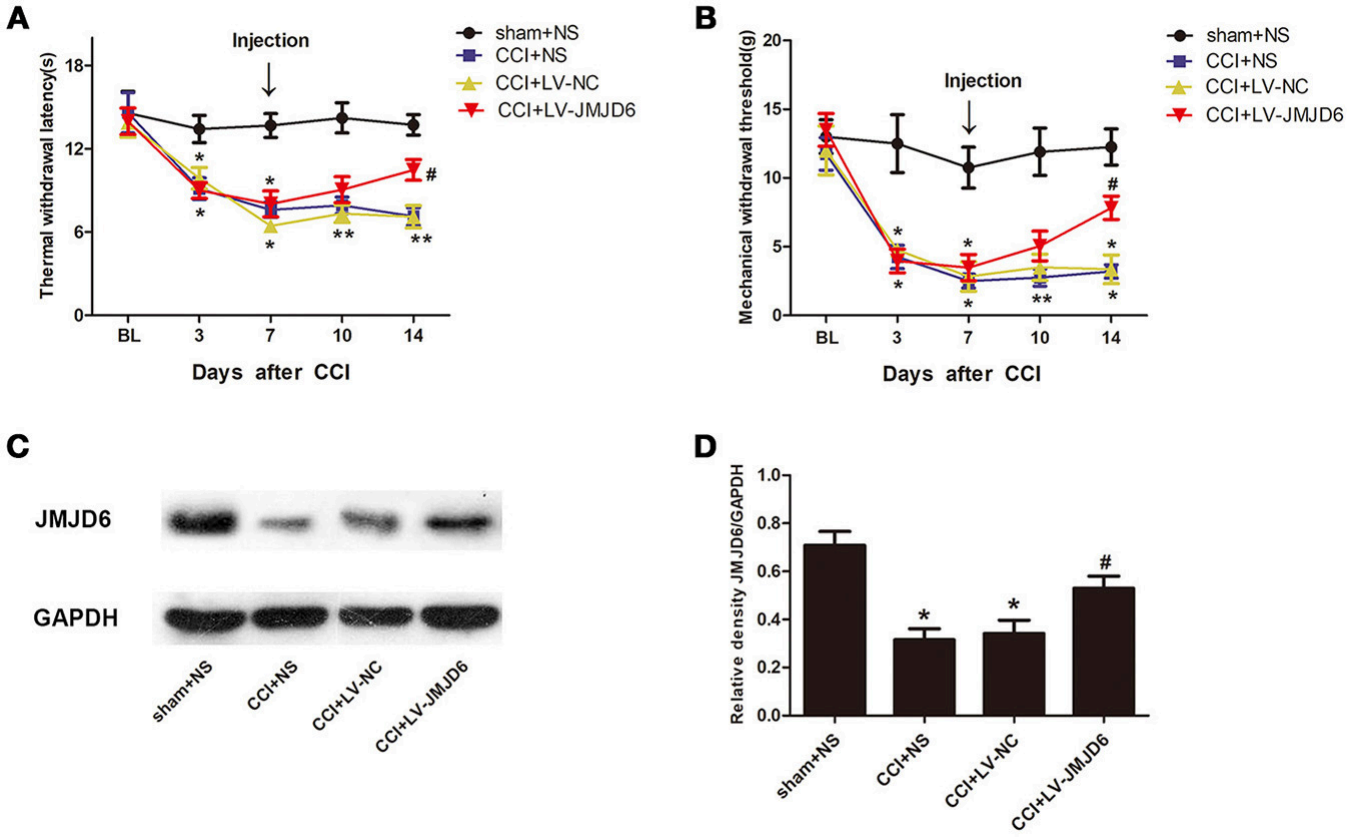
Thermal and mechanical hyperalgesia following CCI is suppressed in rats following intrathecal injection of LV-JMJD6. **(A,B)** Dynamic changes in TWL and MWT (mean ± SEM, *n* = 4). **(C,D)** JMJD6 is increased in the spinal cord after intrathecal injection of LV-JMJD6 in CCI rats (*n* = 4). *p < 0.05, CCI + LV-JMJD6 vs. sham + NS group; ^#^*p* < 0.05, CCI + LV-JMJD6 vs. CCI + NS and CCI + LV-NC groups.

Prior to lentiviral injection, a reduction in TWL and MWT in each CCI rat was observed at day 3 post-CCI when compared with the sham + NS group (*p* < 0.05). The intrathecal injection of LV-JMJD6 not only restored the JMJD6 expression but also attenuated the CCI-induced decrease in TWL and MWT on day 14 post-CCI surgery (CCI + LV-JMJD6 vs. CCI + LV-NC, *p* < 0.05; CCI + LV-JMJD6 vs. CCI + NS, *p* < 0.05; Figures 4A,B). Consistent with the results in Figure 1, the expression of the JMJD6 protein in western blot was decreased in the CCI + NS and CCI + LV-NC groups in comparison to the sham + NS group (Figures 4C,D). There were no significant differences in JMJD6 protein expression between the CCI + NS and CCI + LV-NC groups (*p* > 0.05). In contrast, intrathecal injection of LV-JMJD6 normalized this downregulated expression of JMJD6 protein in CCI rats on day 14 post-CCI (CCI + LV-JMJD6 vs. CCI + LV-NC, *p* < 0.05; CCI + LV-JMJD6 vs. CCI + NS, *p* < 0.05; Figures 4C,D.

### JMJD6 may exert its function by regulating HIF-1α in CCI rats

**Figure 5:**
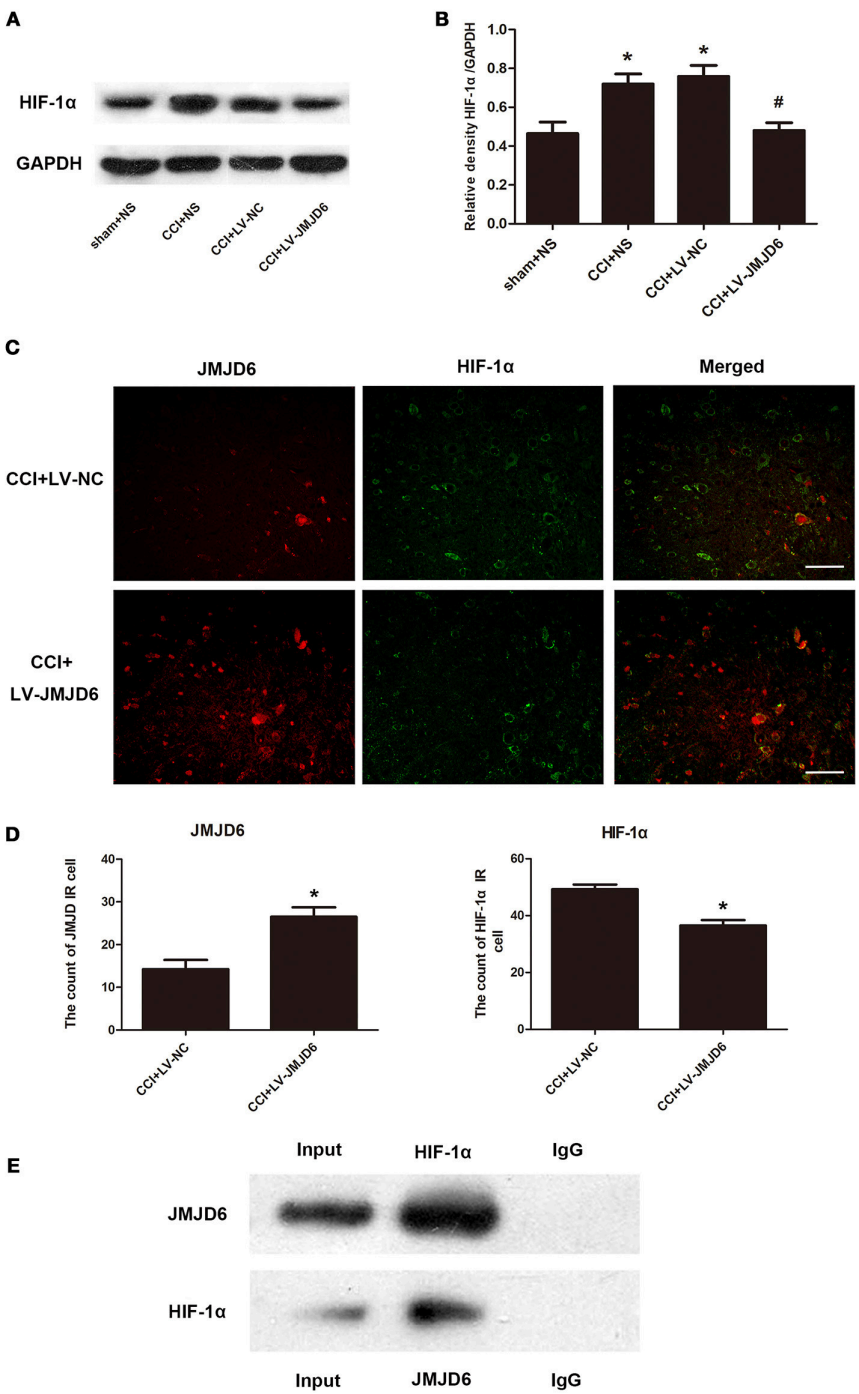
JMJD6 may exert its function in neuropathic pain by regulating HIF-1α. **(A,B)** HIF-1α is decreased in the spinal cord after intrathecal injection of LV-JMJD6 following CCI surgery (*n* = 4). ^#^*p* < 0.05, CCI + LV-JMJD6 vs. CCI + LV-NC group; *p < 0.05, CCI + LV-JMJD6 vs. sham + NS group. **(C)** Double immunofluorescence labeling for JMJD6 (red) and HIF-1α (green) in the rat spinal dorsal horn (L4–6 spinal cord on day 14 post-CCI). Scale bars = 50 μm. **(D)** Quantitative estimation of JMJD6 and HIF-1α in immunofluorescence images. Immunoreactive cells were counted and illustrated (*n* = 4). *p < 0.05, CCI + LV-JMJD6 vs. CCI + NS and CCI + LV-NC groups. **(E)** Co-immunoprecipitation of JMJD6 and HIF-1α in PC12 cells. Immunoprecipitation of JMJD6 followed by western blot for HIF-1α, and immunoprecipitation of HIF-1α followed by western blot for JMJD6.

Sequence analyses have suggested that JMJD6 is a homolog of the HIF asparaginyl hydroxylase (Semenza, 2000), indicating a potential interaction between JMJD6 and HIF-1α. The expression of HIF-1α protein was significantly higher in the CCI + NS and CCI + LV-NC groups compared to the sham + NS group (*p* < 0.05; Figures 5A,B). Intrathecal injection of LV-JMJD6 decreased the expression of HIF-1α protein in CCI rats on day 14 post-CCI (CCI + LV-JMJD6 vs. CCI + LV-NC, *p* < 0.05; CCI + LV-JMJD6 vs. CCI + NS, *p* < 0.05; Figures 5A,B). There was no significant difference in the expression of HIF-1α protein between the CCI + NS and CCI + LV-NC groups (*p* > 0.05).

Double immunofluorescence staining of JMJD6 and HIF-1α in the spinal cord was performed on day 14 post-CCI (Figures 5C,D). There was a significant increase in JMJD6 immunoreactivity and a concomitant decrease in HIF-1α immunoreactivity in the CCI + LV-JMJD6 group in comparison to the CCI + LV-NC group. Further experiments demonstrated a direct interaction between HIF-1α and JMJD6 proteins, as shown by the co-immunoprecipitation of both proteins (Figure 5E).

### Intrathecal injection of LV-JMJD6 decreased the expression of caspase-3

**Figure 6:**
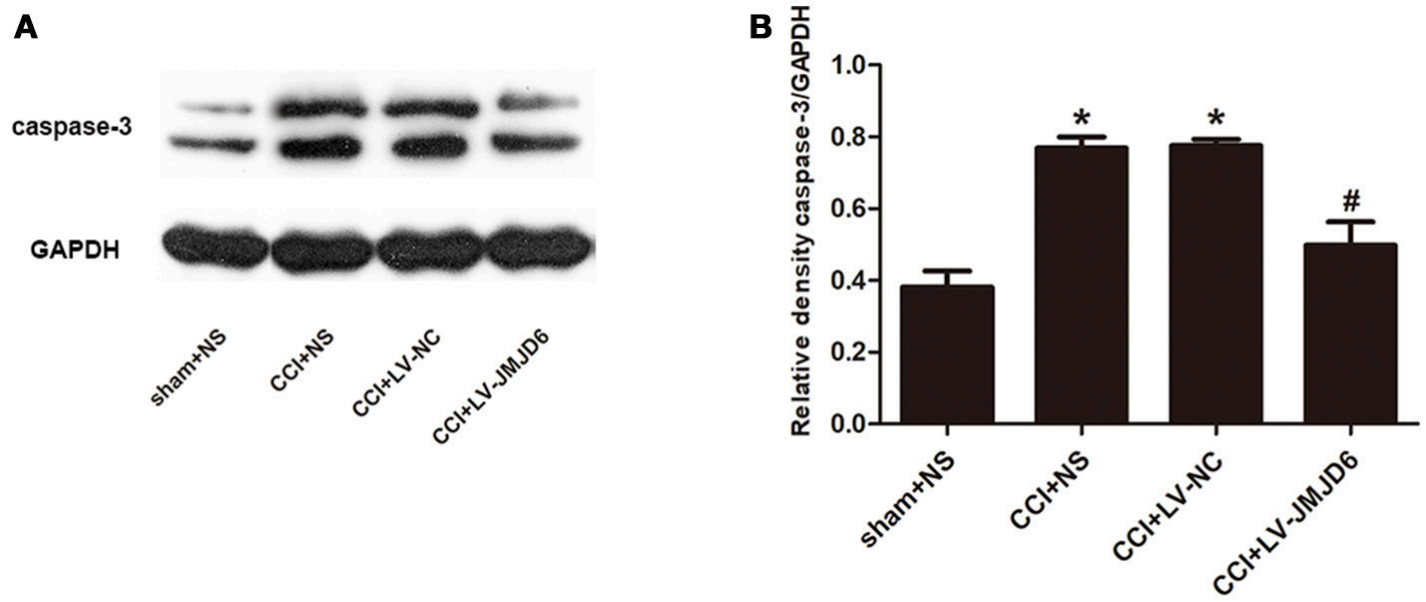
Caspase-3 is decreased in the spinal cord following CCI surgery and intrathecal injection of LV-JMJD6 (*n* = 4). **(A)** Expression of protein Caspase-3 in the lumbar spinal cord in all rats. **(B)** The mean (±SEM) of integrated optical density of Caspase-3/GAPDH in all rats on protein level. *p < 0.05, CCI + LV-JMJD6 vs. sham + NS group; ^#^*p* < 0.05, CCI + LV-JMJD6 vs. CCI + NS and CCI + LV-NC groups.

Our results have indicated that JMJD6 may have a function in neuropathic pain by regulating HIF-1α in CCI rats. Next, we assessed the expression of caspase-3, which is a target gene of HIF-1α (Van Hoecke et al., 2007; Chen et al., 2009). Compared with the sham + NS group, the expression of caspase-3 protein was increased in the CCI + NS and CCI + LV-NC groups (Figures 6A,B). Furthermore, intrathecal injection of LV-JMJD6 decreased the expression of caspase-3 protein in CCI rats on day 14 post-surgery (CCI + LV-JMJD6 vs. CCI + LV-NC, *p* < 0.05; CCI + LV-JMJD6 vs. CCI + NS, *p* < 0.05). There was no significant difference in caspase-3 protein expression between the CCI + NS and CCI + LV-NC groups (*p* > 0.05).

## Discussion

Abnormal excitability of the nervous system following peripheral nerve injury, especially in the nociceptive spinal dorsal horn, is an important mechanism of NPP (Woolf and Salter, 2000). JMJD6 is an evolutionarily conserved and widely expressed nuclear protein (Bose et al., 2004; Cui et al., 2004; Hahn et al., 2008, 2010); however, few studies have reported the localization of JMJD6 in the nervous system. The spinal cord dorsal horn is a major site of nociceptive signal processing (Melzack and Wall, 1965); therefore, we chose this site to observe the temporal distribution of JMJD6 following CCI surgery. Our results show a significant reduction of JMJD6 in CCI rats. We found that JMJD6 is primarily localized to neurons. Many studies have shown that spinal astrocytes are important in NPP (Gwak and Hulsebosch, 2009; Ikeda et al., 2012; Chen et al., 2014; Old et al., 2015), and miscommunication between neurons and glial cells, such as astrocytes and microglia, may also play a part in NPP (Chiang et al., 2012; Burke et al., 2016; Nascimento et al., 2016); however, we did not observe any significant JMJD6 localization to astrocytes or microglia. In this study, we conclude that JMJD6 does not localize to astrocytes or microglia. This is derived only from morphological evidence, which is one of the limitations of our study. Unfortunately, there is no better methodology to examine this.

The intrathecal injection of LV-JMJD6 normalized the expression of JMJD6 protein in the CCI + LV-JMJD6 group on day 14 post-surgery. Furthermore, this normalization led to an attenuation of the CCI-induced reductions in TWL and MWT. Our results indicate that overexpression of JMJD6 could relieve the pathologic process of NPP; however, the signaling pathway associated with this is yet to be elucidated. Different possibilities can be taken into consideration. JMJD6 exerts its inhibitory function through the demethylation of H4R3me2a, and the level of histone methylation influences the synaptic plasticity in the nervous system (Epstein et al., 2001; Ikeda et al., 2012; Na et al., 2015; Aprelikova et al., 2016; Ding et al., 2017). Furthermore, histone demethylation may support a reversal of the pathologic processes in NPP (Semenza, 2000).

JMJD6 is structurally homologous to the HIF asparaginyl hydroxylase, which may take part in the regulation of HIF-1α expression (Semenza, 2000; Alahari et al., 2015). Previous studies have shown that under normoxic conditions, JMJD6 regulates HIF-1α *via* interaction with the von Hippel-Lindau protein, which mediates proteasomal degradation when specific proline residues of HIF-1α are hydroxylated (Maxwell et al., 1999; Epstein et al., 2001; Alahari et al., 2015). HIF plays a vital role in the mammalian hypoxic response; as levels of the HIF-1α subunit increase, dimerization occurs with the β subunit of HIF-β, which activates gene expression (Jayatunga et al., 2014). It has been reported that HIF-1α is overexpressed following CCI surgery, and this overexpression is suppressed following low-level laser therapy, which is in agreement with previous results (Hsieh et al., 2012). In addition, HIF-1α is protective against acute pain following ongoing activation of injured neurons (Kanngiesser et al., 2014; Wu et al., 2015). Our results show that increased expression of HIF-1α protein induced by CCI surgery is reversed following LV-JMJD6 administration, indicating a connection between JMJD6 and HIF-1α in NPP.

The HIF-1 heterodimer binds to the hypoxia response element, which contains the consensus nucleotide sequence 5′-RCGTG-3′ (R: A/G) of target genes to activate transcription (Semenza et al., 1996; Chilov et al., 1999). In silico analysis of the JMJD6 promoter has revealed the presence of three putative HREs of the consensus motif ACGTG within the promoter region of the gene, suggesting that the coactor of the HIF-1α and JMJD6 promoter can change the transcription of JMJD6 and then trigger a cascade reaction of its downstream factors (Semenza, 2000; Hewitson et al., 2002; Chang et al., 2007; Alahari et al., 2015). The present study shows co-immunoprecipitation of JMJD6 and HIF-1α; therefore, we speculate that there is a direct interaction between these proteins.

Caspase-3 is a target gene of HIF-1α (Van Hoecke et al., 2007; Chen et al., 2009). HIF-1 functionally binds to the caspase-3 promoter after photothrombotic cerebral ischemia (Van Hoecke et al., 2007). Furthermore, HIF-induced modulations of caspase-3 have been observed in the hippocampus of rats following transient global ischemia, and inhibition of caspase-3 reduces neuronal loss and brain edema (Liu et al., 2016). In our study, LV-JMJD6 administration normalized JMJD6 and decreased both HIF-1α and caspase-3 protein expression. These results strengthen our hypothesis that JMJD6 may exert its functions by regulating HIF-1α in this rat model of NPP.

In conclusion, we have reported the expression and localization of JMJD6, and its potential relationship with HIF-1α in an NPP model. JMJD6 protein expression was reduced in rats after CCI surgery, which was accompanied by significant behavioral changes associated with NPP. We found in rats exposed to CCI that JMJD6 was primarily expressed in the nuclei of neurons in the gray matter of the dorsal horn and may exert its functions in NPP by regulating HIF-1α. These data contribute to our understanding of the role of JMJD6 and HIF-1α in the pathogenesis of NPP.

## Author contributions

XZ and QG designed and conceived the experiments. CM, MX, and CW performed the experiments. WZ and QG analyzed the data. CM, RC, and CH contributed reagents, materials and analytical tools. CM and XZ wrote the manuscript.

### Conflict of interest statement

The authors declare that the research was conducted in the absence of any commercial or financial relationships that could be construed as a potential conflict of interest.
